# The Season for Peace: Reconciliation in a Despotic Species (*Lemur catta*)

**DOI:** 10.1371/journal.pone.0142150

**Published:** 2015-11-16

**Authors:** Elisabetta Palagi, Ivan Norscia

**Affiliations:** 1 Natural History Museum, University of Pisa, Calci, Pisa, Italy; 2 Institute of Cognitive Sciences and Technologies, Unit of Cognitive Primatology & Primate Center, CNR, Rome, Italy; Northern Illinois University, UNITED STATES

## Abstract

However despotic a social group may be, managing conflicts of interest is crucial to preserve group living benefits, mainly based on cooperation. In despotic groups, post-conflict management via reconciliation (the first post-conflict reunion between former opponents) can occur, even if conciliatory rates are considerably different. *Lemur catta* is defined as a despotic species because groups are characterized by a strict linear hierarchy maintained by the adult females (the dominant sex) mainly via aggression. Reconciliation was reported in one out of four captive groups of *L*. *catta*. Here we investigate which variables influence the occurrence of reconciliation in these despotic groups. We analyzed 2339 Post Conflict (PC)-Matched Control (MC) observation pairs, collected on eight groups (five in the Berenty forest, Madagascar; three hosted at the Pistoia Zoo, Italy). Since *L*. *catta* is characterized by steep female dominance but shows female-female coalitionary support, we expected to confirm the presence of reconciliation in the study species. Consistently, we found reconciliation in one captive group and two wild groups, thus providing the first evidence of the presence of this phenomenon in wild *L*. *catta*. Moreover, because this species is a seasonal breeder (with mating occurring once a year), we expected seasonal fluctuations in reconciliation levels. Via a GLMM analysis using data from all wild groups and on a captive group followed for more than one year, we found that season (but not rank; individuals’ identity, sex, and age; or group identity) significantly affected individual reconciliation rates, and such rates were lowest during the mating period. Thus, reconciliation can be present in groups in which dominants strongly influence and limit social relationships (steep dominance hierarchy) except when the advantages of intra-group cooperation are overcome by competition, as occurs in seasonal breeders when reproduction is at stake. We conclude that in despotic social groups in which coalitions are observed, the right question is not *if* but *when* reconciliation can be present.

## Introduction

The management of conflicts of interest is crucial to preserve group living benefits, even in despotic societies. In these kinds of societies, to preserve social integrity, violence is minimized via the strict control exerted by dominants over other individuals (“negative peace”, *sensu* Galtung [1]). Yet, in humans and other social mammals, dominant individuals or subgroups may need the support of others to obtain resources and maintain the *status quo* [2–7]. Consequently, strategies of mutual help other than competition for dominance and resources must be enabled, such as cooperative breeding, hunting, and coalitionary support during between-group conflicts [8–11].

Reconciliation or peace-making, defined as the first affinitive contact between former opponents occurring within few minutes after the conflict, is one of the main mechanisms to manage conflicts [12]. The phenomenon is present in social animals, including a bird species (e.g. ravens, *Corvus corax* [13]), various non primate mammals (e.g. domestic goats, *Capra hircus* [14]; dolphins, *Tursiups troncatus* [15]; domestic dogs, *Canis lupus familiaris* [16]; horses, *Equus caballus* [17]; red-necked wallabies, *Macropus rufogriseus* [18]), and human and non human primates (*Homo sapiens* [19]; chimpanzees, *Pan troglodytes* [20], [21]; bonobos, *Pan paniscus* [22]; *Gorilla beringei* and *Gorilla gorilla* [23–25]; wild macaques, *Macaca* spp. [26, 27]; captive guereza, *Colobus guereza* [28]; captive patas monkeys, *Erythrocebus patas* [29]; captive squirrel monkeys, *Saimiri sciureus* [30]; captive white-faced capuchins, *Cebus capucinus* [31]).

By restoring the relationship between former opponents [32–39], reducing the probability of further fights [23], [33], [34], [40–45] and/or reducing anxiety in the victim [21], [46–50], reconciliation is crucial to preserving social unity from the disruption caused by uncontrolled conflict spreading in the group. Therefore, reconciliation is expected to be present any time that it is valuable for the group members (including dominants) to preserve the alliances that facilitate group survival, thus preserving the benefits of group living [51].

Consistently, reconciliation has been found also in species with a despotic dominance style [5; 52–55]. According to the definition of Flack and de Waal [52], in despotic groups dominance dyadic asymmetries remains quite stable over time because they reinforced through severe aggression. Instead, in tolerant groups dyadic asymmetries can exist but many relationships are unresolved. Examples of animals living in despotic groups and that are able to reconcile include spotted hyenas (*Crocuta crocuta* [53]), wolves (*Canis lupus lupus* [5]), Japanese macaques (*Macaca fuscata* [54]), and wild chacma baboons (*Papio ursinus*: Cheney, Seyfarth & Silk [55]). Similar to these species, *Lemur catta* can be defined as despotic because groups are characterised by a linear and steep hierarchy with clear-cut dominance relationships [56]. Females are dominant and their dominance is maintained also through severe aggression by dominants over subordinates [56–63]. In this species, the presence of reconciliation was found in one out of four captive troops in which post-conflict management was studied [64],[65].

The linkage between reconciliation and the level of authoritativeness (or despotism) has been qualitatively examined in humans, with friendly peacemaking being favored by minimal authority (power exercised over others; [66]). The linkage between reconciliation and dominance style has been also quantitatively assessed in tolerant to despotic macaque species ([52],[67]), with tolerant species (e.g. Tonkean macaques, *Macaca tonkeana* [37],[68], [69–71]) showing higher reconciliation levels than despotic species (Japanese macaques, *Macaca fuscata* [54]). The same linkage has been hypothesized in strepsirrhine primates [64], which can also show more or less mild and flexible dominance hierarchies [56]. In this primate taxon, reconciliation was indeed found in species with more relaxed (i.e. less steep or transitive) dominance relationships (captive *Eulemur* wild *Eulemur rufusxcollaris* [45]) *rufus* [64], [72]; wild *Propithecus verreauxi* [73]) but not in captive *Eulemur macaco* showing strong female dominance [72].

In the present study, we investigate the factors that can explain the occurrence of reconciliation (or lack thereof) in different captive and wild groups of *L*. *catta* and make inferences about the conditions that favor the presence of reconciliation in despotic groups. As a primate species belonging to the group (strepsirrhines) that diverged from the common ancestor some 60 million years ago [74], *L*. *catta* also offers the possibility to make inferences about the biological roots of peace-making dynamics found in humans and all other primates. For this investigation, we analyzed the data collected on the focal species both in the wild and in captivity across more than a decade to verify the following predictions:

### Prediction 1

Similar to wolves and hyenas [5], [75], [76] *L*. *catta* is characterized by rigid hierarchy and high competition levels [57–63], [77–79]. Analogous to ring-tailed lemur troops, packs (in the case of wolves; [80]) and clans (in the case of hyenas [81], [82]) strictly defend their territories by directing severe aggression towards potential immigrants. Finally, although in a more limited form compared to canids and hyenids, *L*. *catta* females (the dominant sex in this species) are able to form coalitions, especially against other females, to preserve their dominance status or to gain the possibility to use a territory [10]. These traits led us to predict that, as in other despotic but cooperative species [58], reconciliation may be present in *L*. *catta* not only in captivity but also in the wild.

### Prediction 2

In the animals breeding once or twice in the year, seasonality strongly affects social behaviour and competition levels [83]. Majolo & Koyama [84] found that in the population of despotic *Macaca fuscata* from Yakushima Island reconciliation levels changed seasonally. As most lemur species, *L*. *catta* lives and has evolved in a highly seasonal environment [61], [85,86] and is a seasonal breeder [58]. In fact, females are receptive once a year [87–89] and the mating period (from three weeks to two months depending on the site and the definition; see also: [57], [58], [90], [91]) is characterised by high competition and low affiliation levels. During the mating period, competition within and between sexes is extremely high and affiliation levels are low [58], [77], [92], [91]. Therefore, we expected that in *L*. *catta* seasonality would particularly affect reconciliation levels.

## Methods

### Ethics statement

Since the study was purely observational the Animal Care and Use board (University of Pisa) waived the need for a permit. The study was conducted with no manipulation of animals. The study was carried out in the private Reserve of Berenty (South Madagascar) and at the Pistoia Zoo (Pistoia, Italy). De Heaulme and family, owners of Berenty and Mr Cavicchio, owner and director of the Pistoia Zoo, permitted us to observe animals.

### Study species

*Lemur catta* (ring-tailed lemur) is a cathemeral species characterized by seasonal fluctuations in olfactory behavior, group dispersal, tolerance level, and reproduction [58], [78], [79], [93–97]. *Lemur catta* has a steep, consistent, highly transitive and cohesive hierarchy (*sensu* Norscia and Palagi [56]), with females dominant over males [58], [59], [78], [98–99]). Male hierarchy is unstable, and at times, non-transitive, and both female-female and male-male dominance hierarchies are fluid and can change over time [100–102].

The mating season overlaps among the different groups of a population and can last from three weeks to two months (depending on the site, the year, the definition of mating period; [57], [58], [90], [91], [103]. However, the onset of the mating period varies between groups, and the whole mating season for the lemur population spans up to four months [57],[103]. Females experience an annual estrus of a few hours to days, and receptivity lasts 10–24 h after which the estrus period ends [58], [87]-[89], [59]. A second or third belated estrus is possible [58], [78], [79]). *Lemur catta* females have a visible estrus, which may be asynchronous with other females in their group [104]. The mating period starts about one month before copulations, when female perineal area starts to enlargen and the center of the genitalia becomes larger and pinker: this period of swelling anticipates estrus [58], [87]. Generally, receptivity coincides with the last day of maximal pink coloration of vaginal labia ([87], [103].

### Study location and subjects

#### Berenty (Madagascar)

We conducted this research on wild lemurs in the gallery forest of Berenty, a reserve on the Mandrare River in Southern Madagascar (for an extensive description of the forest, see [105]). Data collection was conducted in the northern part of the forest called Ankoba (S 24.998; E 46.298), a 40-ha secondary forest 50- to 60-years-old, with canopy at 10–15 m (except for few emergent acacias to more than 20 m) and high lemur density [105]. Observations were carried out in the periods November 2006-February 2007, April-July 2008, and March-April 2011 on five troops of *L*. *catta*. Details on group composition and observation periods are reported in Table 1. Kin relationships among group members were unknown but groups at Berenty (and other sites) are largely female matrilines (including sibling and offspring of the alpha female [10],[59], [106], [107]. The individuals were well habituated to the presence of humans. As in previous studies, individual identification was based on sex and distinctive external features [56–58].

**Table 1:** Composition of wild and captive groups, observation n periods and study sites.

Group	Observation months	Period	Males_adult_	Females_adult_	Males_juvenile_	Females_juvenile_	Study site
							WILD
A_w_	Nov_2006_-Feb_2007_	Lactation	4	4	1	0	Berenty
B_w_	Nov_2006_-Feb_2007_	Lactation	4	6	2	1	Berenty
C_w_	May-Jul_2008_	Pregnancy	3	6	1	2	Berenty
D_w_	Apr-Jun_2008_	Mating	6	8	1	3	Berenty
E_w_	Mar-Apr_2011_	Premating	5	5	5	2	Berenty
							CAPTIVITY
A_c_	Feb-Mar_1999_	Pregnancy	2	3	0	0	Pistoia
B_c_	Apr-May_1999_	Lactation	2	4	2	0	Pistoia
C_c_	Nov_2003_-Feb_2005_	Premating, Mating, Lactation, Pregnancy	4	4	0	2	Pistoia

#### Pistoia Zoo (Italy)

We studied three captive troops (here named A, B, and C) at the Pistoia Zoo (Italy) in the periods February-May 1999, November 2003-February 2005. Details on group composition and observation periods are reported in Table 1. The captive groups were largely composed by the alpha female and kin (siblings and offspring of the alpha female). The lemurs were housed in an outside grassy enclosure (98 m^2^). In 1999, groups A and B were kept in two separated indoor halls on the coldest days of the year (A: 10 m^2^ indoor facility; B: 20 m^2^ indoor facility). Large glass windows in the two indoor facilities allowed the lemurs to follow the natural day-light 24-h cycle. Each group utilized the outside enclosure for 4–6 h per day, separately. In 2003–2005, another group (C_c_) was hosted at the zoo and could use the indoor facility previously used by the other groups (not present anymore). The observations took place outdoors and lasted from the end of October 2003 to February 2015. As in the wild and in previous studies at Pistoia Zoo, individual identification in captivity was based on sex and distinctive external features [57] [65], [73], [74].

### Data collection

Systematic data collection was preceded by a training period that lasted until the data collected by the two observers (on aggression and affiliation behavioral patterns) matched in 95% of cases [108]. The excellent visibility condition of the Berenty forest allowed us to apply the same protocol to the wild as was used in captivity. For each agonistic encounter we recorded: (1) identity of the two opponents; (2) aggressive behavioral patterns (mainly chase, bite, grab, jump); and (3) submissive/frightened patterns (flee and vocalization). The agonistic interaction was labeled as “decided” when one of the two opponents gave up the fight (by retreating, fleeing or running away) and the winner could be therefore determined with certainty. For a comprehensive ethogram see [109].

After the last aggressive pattern of any given agonistic event, we followed the loser of the interaction (as the focal individual) for a 10 min post-conflict period (PC). Matched control observations (10 minute long MCs) took place during the next possible day at the same time, context (feeding, resting or travelling) and physiological season (lactation, pre-mating, mating, and pregnancy; see details below) as the original PC. MC data were collected only if all these conditions were met. The MC was conducted on the same focal animal, in the absence of agonistic interactions during the 10 min before the beginning of the MC and when the opponents had the opportunity to interact, within a distance of 10 m maximum [110], [111]).

We considered four groups of affinitive behaviors to identify the first conciliatory contact: body contact (body-to-body contact excluding tails, huddle); greeting (naso-nasal, face grooming); grooming (unidirectional, reciprocal or mutual); olfactory contact (sniffing body, sniffing genitals, and skin licking) [109]). Proximity was not considered because it does not necessarily indicate affiliation. We collected a total of 2339 PC-MC (1461 in captivity and 878 in the wild). For both PCs and MCs we recorded: (1) starting time; (2) type of first affinitive interaction; (3) time of first affinitive contact; (4) partner identity.

### Operational definitions and data analysis

Reconciliation analysis was carried out at the individual level, taking the recipient of the aggression as the individual of reference. For each animal we determined the number of attracted, dispersed and neutral pairs over all PC-MC pairs. In attracted pairs, affinitive contacts occurred earlier in the PC than in the MC (or they did not occur at all in the MC), whereas in dispersed pairs the affinitive contacts occurred earlier in the MC than in the PC (or they did not occur at all in the PC). In neutral pairs, affinitive contacts occurred during the same minute in the PC and the MC, or no contact occurred in either the PC or the MC [110].

Due to the small sample size and/or deviation from normality (Exact Kolmogorov-Smirnov, p<0.05) we used the Exact Wilcoxon signed-ranks test [112], [113] to compare attracted versus dispersed pairs. Attracted and dispersed pairs were measured at the individual level, thus ensuring the independency of data points. The pair-wise comparison between attracted and dispersed pairs allows checking whether reconciliation is present (if the number of attracted pairs is significantly higher than the number of dispersed pairs) or not.

In addition to determining whether reconciliation was present or not, we assessed the individual rates of conciliatory tendencies of individuals. The measure of corrected conciliatory tendency (CCT; [114]) allows evaluating the level of individual reconciliation by considering the attracted minus dispersed pairs divided by the total number of PC-MC pairs. Individual CCTs were used to determine the mean CCT in wild and captive conditions.

To assess the effect of the different factors on individual CCTs (scalar, dependent variable), we ran two sets of General Linear Mixed Model (GLMM). The first GLMM was performed on all the study groups (Table 1). As fixed factors, we considered sex (binomial: male/female), age (binomial: juvenile/adult), rank position (scalar), season (multinomial: 1–4), individuals (nominal), and groups (nominal). Due to the inter-independence of sex and age, and sex and rank (because females outrank males and adults outrank subadults), these three factors were entered as two combined variables (sex*rank and age*rank). In order to attempt to remove possible confounding variables, the second GLMM was performed only on groups C_c_ for which data collection had covered all seasons (Table 1). We considered the same fixed factors included in the first GLMM except for group ID.

Since CCT distribution was normal in both cases (Kolmogorov-Smirnov, p = n.s.), an identity link function was used. We tested models for each combination involving the variables of interest, spanning from the null model (only intercept) to the model including all the fixed factors (full model). To select the best model, we used the Akaike’s Corrected Information Criterion (AICc), a measure for comparing mixed models based on the −2 (Restricted) log likelihood. The AICc corrects the Akaike’s Information Criterion (AIC) for small sample sizes. As the sample size increases, the AICc converges to AIC. The model with a lower value of AICc was considered to be the best model. To avoid the increase of type II errors, factors were excluded from a model only if this improved the model fit by >2 AICc units [115]. The value of degrees of freedom is given by the effective sample size (N) minus the rank design matrix of fixed effects (X). The denominator degree of freedom is estimated by SPSS via Satterthwaite’s approximation.

We used all dyadic decided agonistic interactions to prepare a winner/loser socio-matrix and carry out hierarchical rank order analysis, by using MatMan 1.0 based on I&SI rankings (Noldus Information Technology, Wageningen, Netherlands; [116]). To assign the age class to each animal, the individuals were distinguished between adults (regularly performing genital marking, informing an age >18 months) and juveniles (not performing genital marking) [117].

Four seasons were recognized: lactation (1), pre-mating (2), mating (3), pregnancy (4) (The numbers correspond to how the seasons have been entered in the GLMM model). For the captive groups (in the northern hemisphere) the different seasons were: lactating season (group B_c_: April-May 1999; group C_c_: April-August 2004); pre-mating (group C_c_: September-October 2004), mating (group C_c_: November-December 2003; November-December 2004), pregnancy (group A_c_: February-March 1999; group C_c_: January-March 2004; January-February 2005). Individual CCTs for group C_c_ (observed for more than one season) were calculated using the PC-MC collected for each season. In the wild the mating period varied depending on the group (refer to Table 1 for the groups): pre-mating (group E_w_: March-April: 2011), mating (group D_w_: April-May-beginning of June 2008), pregnancy (group C_w_: May-July 2008), and lactating season (groups A_w_ and B_w_: November-February 2006). The mating period began when at least one female of the group started showing genital swelling from about 1.5–3 cm in length and developing a pink center [57], [58]. In a group, the pregnancy was considered as starting after the last copulation day (confirmed ex-post by births) whereas lactation started when a female in the group gave birth. Overall two mating periods were available in captivity and one in the wild.

## Results

A previous study [65] showed that reconciliation was present in captive group A_c_ but not in group B_c_ (Table 1) so those analyses are not reported here. The overall CCT calculated here for the first time for all groups was 10.25% ±2.24 (Mean ±SE). In the wild the CCT was 10.99% ±2.44 and in captivity 9.62% ±3.60 (Mean ±SE). Mean CCT% (±SE) for each group are reported in Table 2.

**Table 2:** Mean Corrected Conciliatory Tendency (CCT %) ± Standard Error (SE) for each study group.

Group	CCT%: Mean±SE
A_w_	19.55±7.52
B_w_	18.62±8.51
C_w_	14.63±6.96
D_w_	5.74±2.72
E_w_	3.69±2.20
A_c_	43.17±19.24
B_c_	-14.83±4.23
C_c_	9.47±6.73

**Fig 1:**
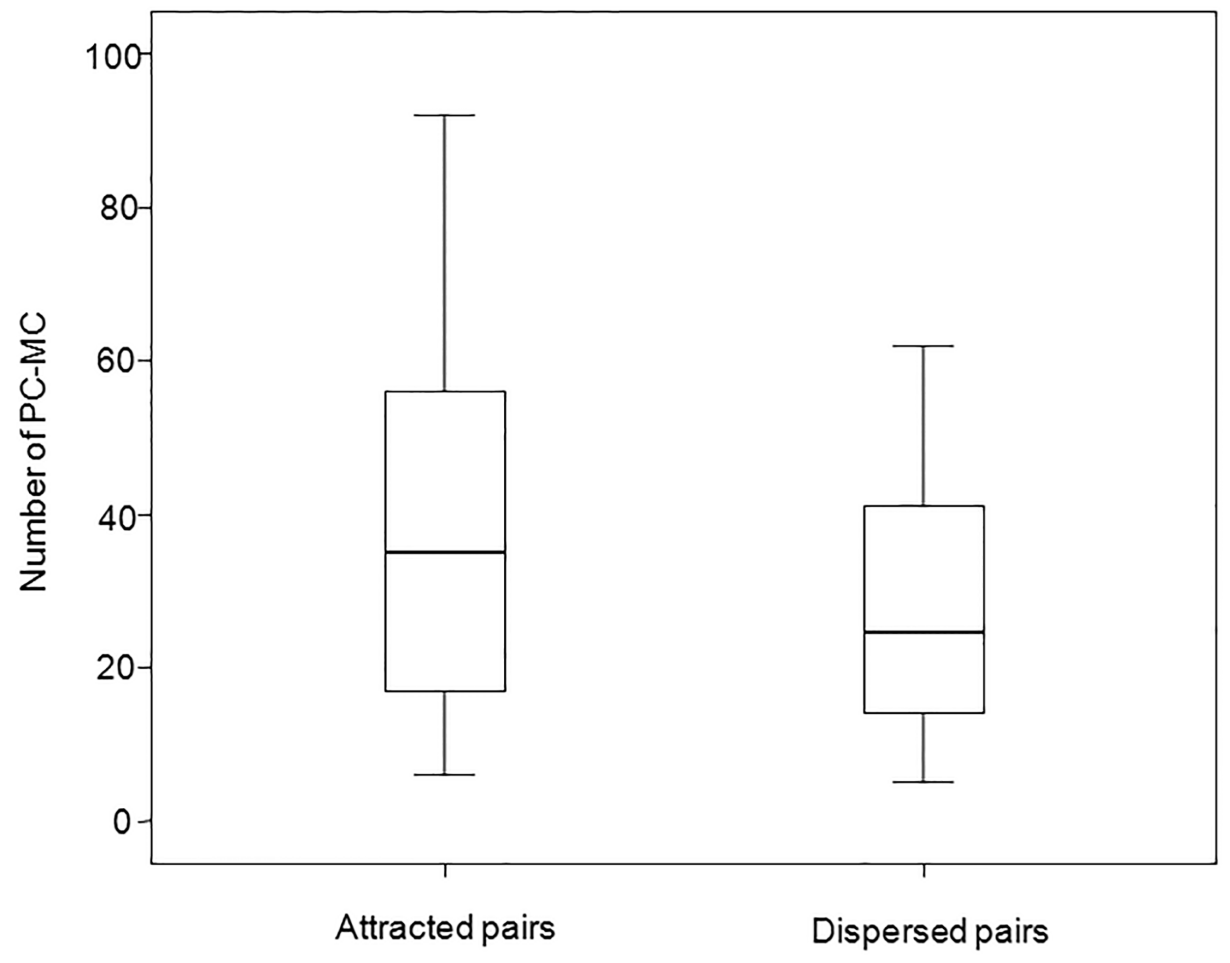
Box plot showing the significant difference (Exact Wilcoxon’s test, p<0.05) between the number of attracted versus dispersed pairs in the *Lemur catta* troop C_c_ (November 2003-February 2005), observed at the Pistoia Zoo (Italy). Solid horizontal lines indicate medians; the length of the boxes corresponds to inter-quartile range; thin horizontal lines indicate range of observed values.

**Fig 2:**
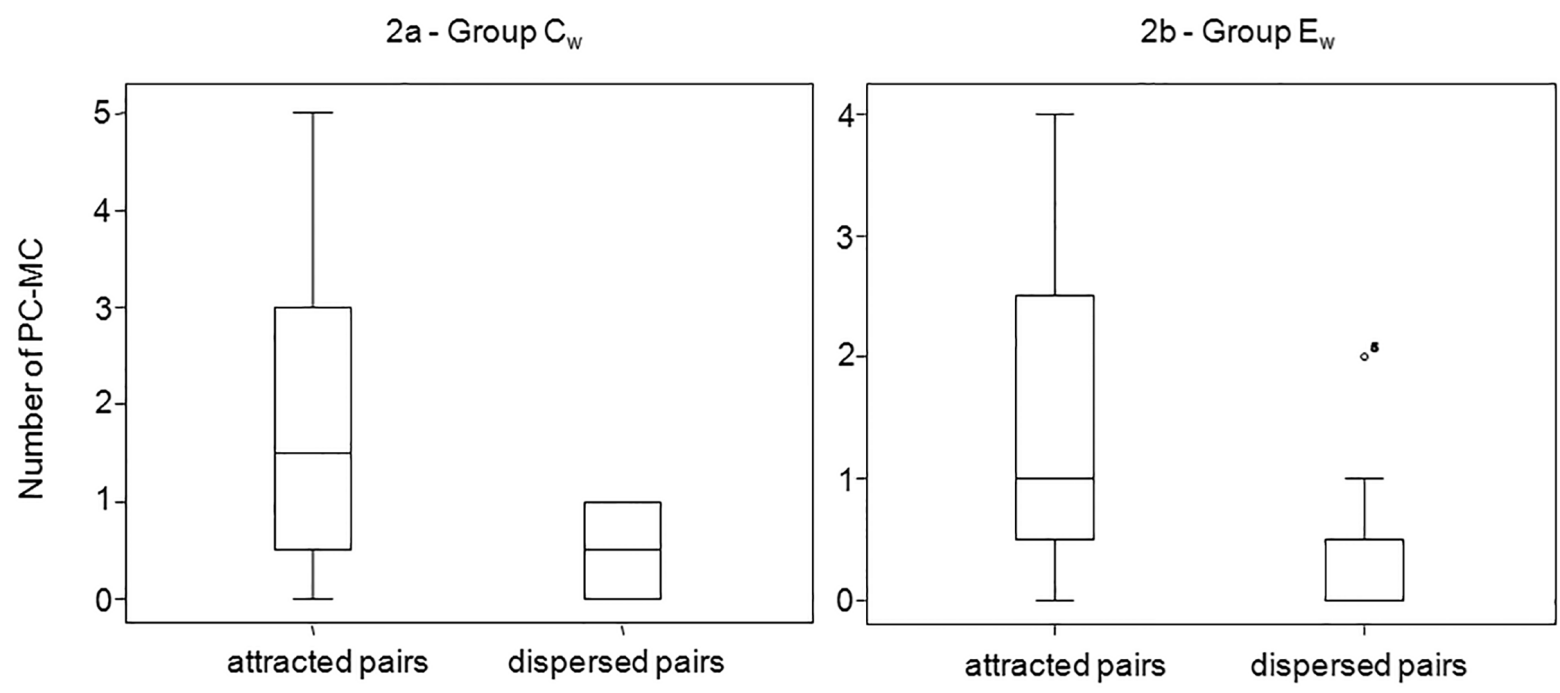
Box plot showing the significant difference (Exact Wilcoxon’s test, p<0.05) between the number of attracted versus dispersed pairs in two wild *Lemur catta* troops (C_w_: May-July 2008, Fig 2a on the left; E_w_: March-April 2011, Fig 2b on the right) observed in the Berenty Forest (Madagascar). Solid horizontal lines indicate medians; the length of the boxes corresponds to inter-quartile range; thin horizontal lines indicate range of observed values.

For captive group C (Table 1) we found a significant difference between the number of attracted pairs (in which affinitive contacts occurred earlier in the PC than in the MC or they did not occur at all in the MC) and the number of dispersed pairs (in which affinitive contacts occurred earlier in the MC than in the PC or they did not occur at all in the PC; attracted>dispersed pairs: T = 5, N = 10, ties = 1, p = 0.004; Fig 1). In the wild, reconciliation was present in two groups out of five (groups C_w_ and E_w_). In fact, we found a significant difference between attracted and dispersed pairs (attracted>dispersed) for group C_w_ (T = 0, N = 12, ties = 6, p = 0.031; Fig 2A) and group E_w_ (T = 2.50, N = 15, ties = 6, p = 0.020; Fig 2B). No significant difference between attracted and dispersed pairs was found for group A_w_ (T = 0, N = 8, ties = 4, p = 0.125), group B_w_ (T = 12, N = 11, ties = 2, p = 0.254) and group D_w_ (T = 19.50, N = 18, ties = 7, p = 0.254).

For both captive and wild settings, the aggression distribution according to the different sex class combination is reported in Table 3 and shows that aggression levels of females toward males and between males were maximum during the mating season. During pregnancy and lactation the majority of conflicts involved females.

**Table 3:** Aggression distribution (%) according to the different sex class combinations for all seasons, in the wild (W) and in captivity (C). Sex class combinations are: ff (females attacking female), fm (female attacking male), mf (male attacking female), mm (male attacking male).

	ff%	fm%	mf%	mm%
**matingC**	11,76	56,62	3,68	27,94
**prematingC**	50	25	12,5	12,5
**pregnancyC**	43,67	38,61	5,7	12,03
**lactationC**	51,78	27,74	3,12	17,6
**matingW**	8,93	65,57	1,1	24,41
**prematingW**	28,55	56,62	0,12	14,7
**pregnancyW**	35,21	40,37	0	23,83
**lactationW**	45,64	41,9	8,23	4,24

**Fig 3:**
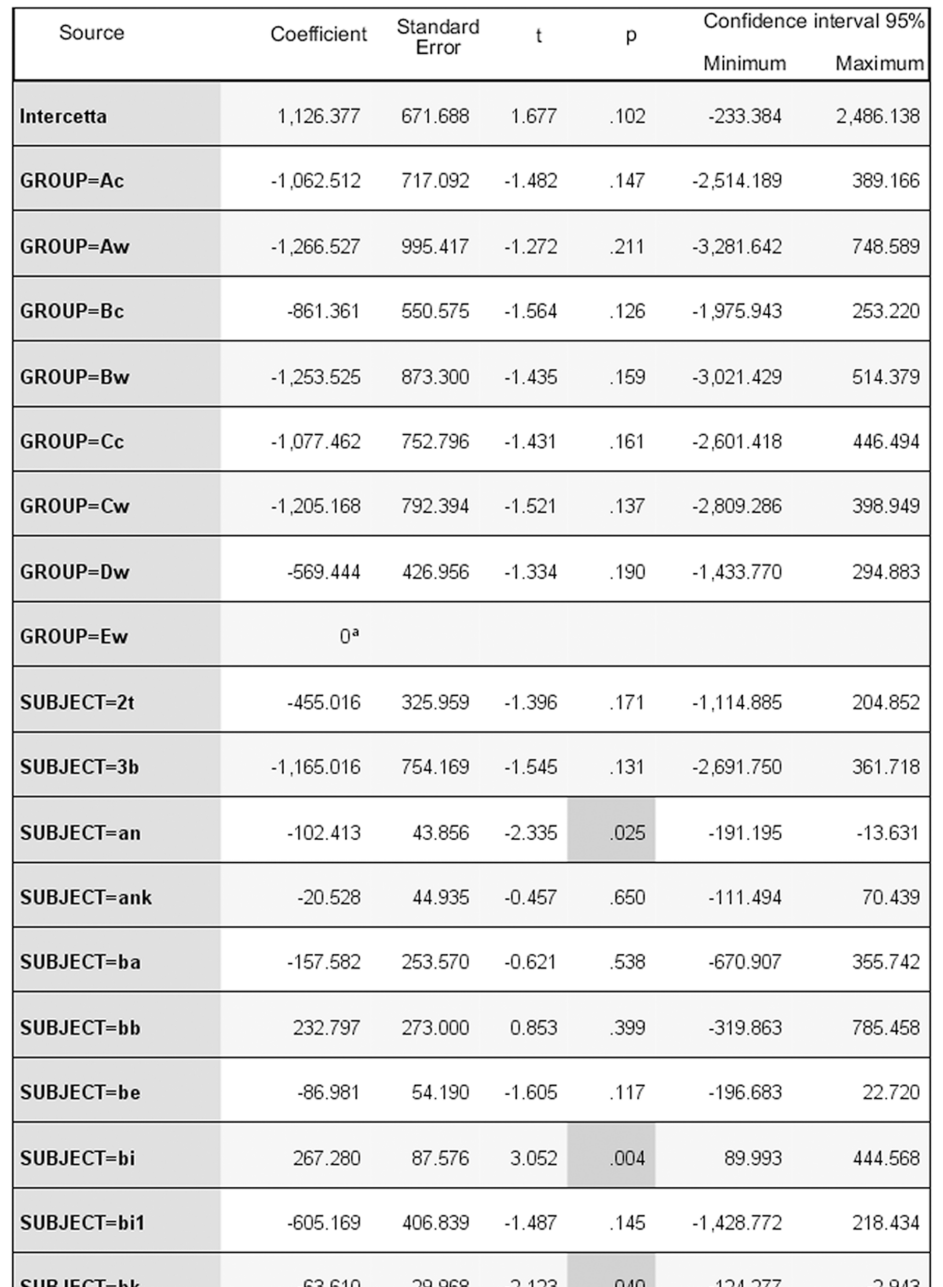
Output of the best model explaining the distribution of Corrected Conciliatory Tendencies (CCT %, scalar target variable) for all the study groups. AICc = 430, 295. Season: 1 = lactation; 2 = pre-mating; 3 = mating; 4 = pregnancy. Sex: 0 = male; 1 = female. Age class: 0 = subadult; 1 = adult. Rank range: 1–16 (rank position is relative to each group). ^a^Redundant coefficients. Please refer to S1 Fig in the Supporting Information for a full size version of Fig 3.

**Fig 4:**
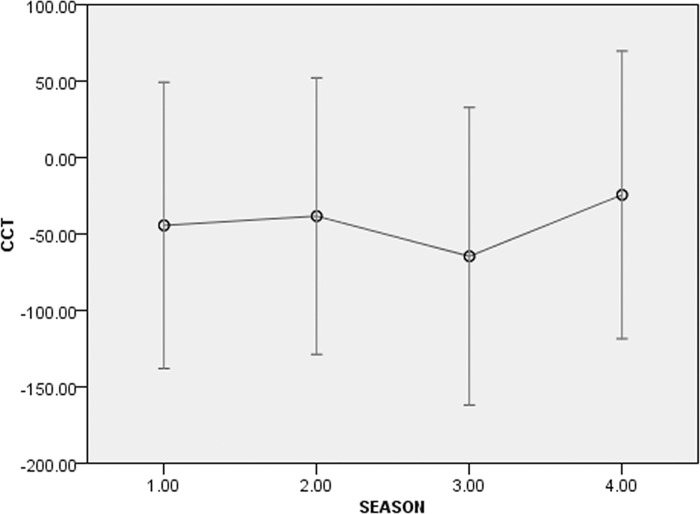
SPSS 20.0 output bar graph showing estimated means of Corrected Conciliatory Tendency (CCT, %) for the significant effect (season: 1 = lactation; 2: pre-mating; 3 = mating; 4 = pregnancy), for all the study groups. Season is the only factor that significantly influences the CCT distribution in the study groups (GLMM; F = 0.718, df1 = 73, df2 = 40, p = 0.890). The conciliatory tendency % is lowest during mating.

Of all the GLMM models tested on all groups (AICc range = 393.675–1107.725) the best one was the full model (Intercept: F = 1.104, df1 = 77, df2 = 38, p = 0.376), including the combination of individual features (sex*rank: F = 1.448, df1 = 1, df2 = 38, p = 0.236; age*rank: F = 0.849, df1 = 1, df2 = 38, p = 0.363), the group identification (F = 1.779, df1 = 1, df2 = 38, p = 0.190), individual identity (F = 0.698, df1 = 64, df2 = 38, p = 0.899), and the season (lactation, pre-mating, mating, and pregnancy; F = 5.282, df1 = 3, df2 = 40, p = 0.004). Fig 3 shows the model output for the best model. Even if part of variability is influenced by individual CCT levels, only the season had a significant effect on the distribution of CCTs, lowest during the mating season (Figs 3 and 4).

**Fig 5:**
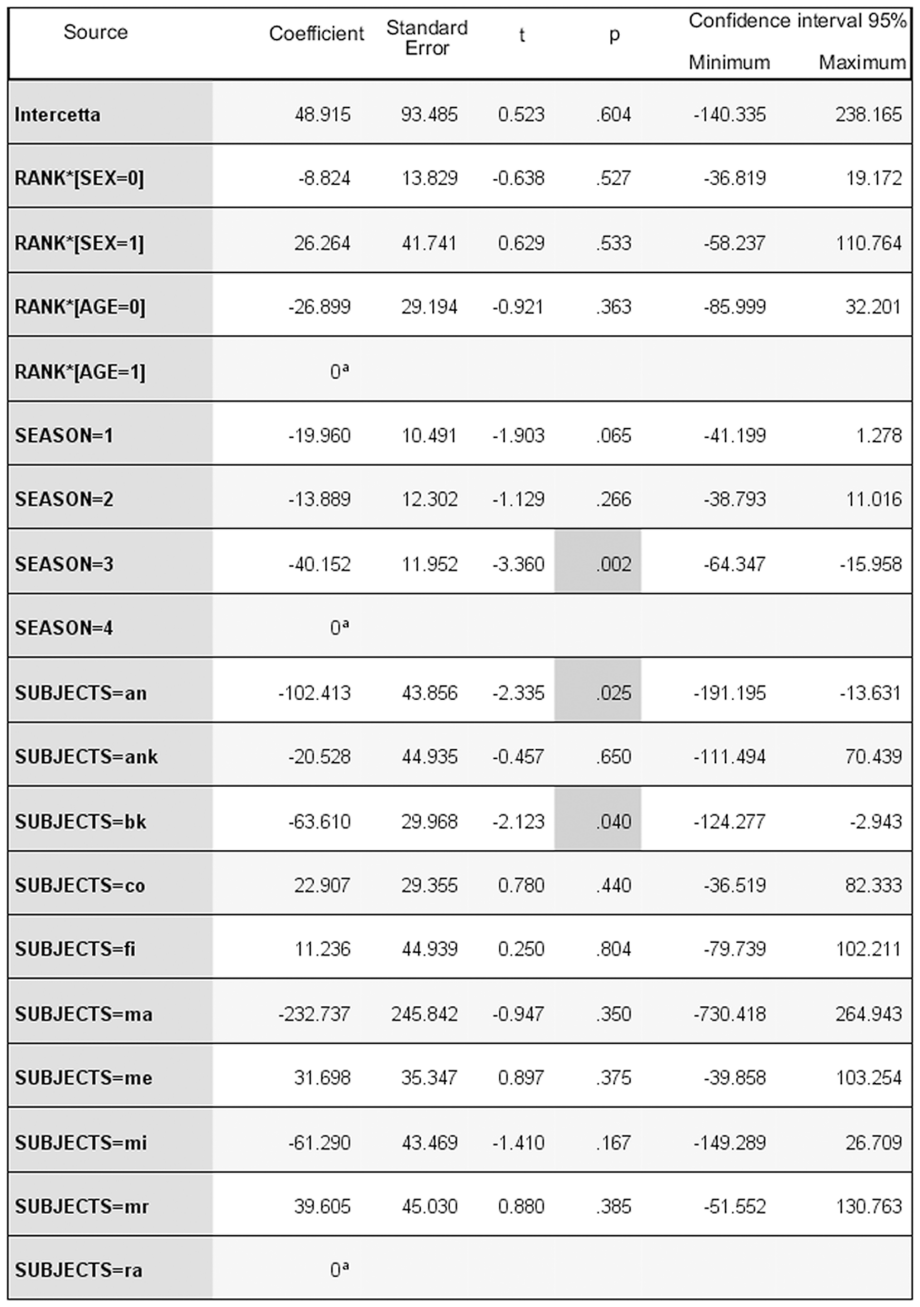
Output of the best model explaining the distribution of Corrected Conciliatory Tendencies (CCT %, scalar target variable) for group C_c_. AICc = 398.767. Season: 1 = lactation; 2 = pre-mating; 3 = mating; 4 = pregnancy. Sex: 0 = male; 1 = female. Age class: 0 = subadult; 1 = adult. ^a^Redundant coefficients.

**Fig 6:**
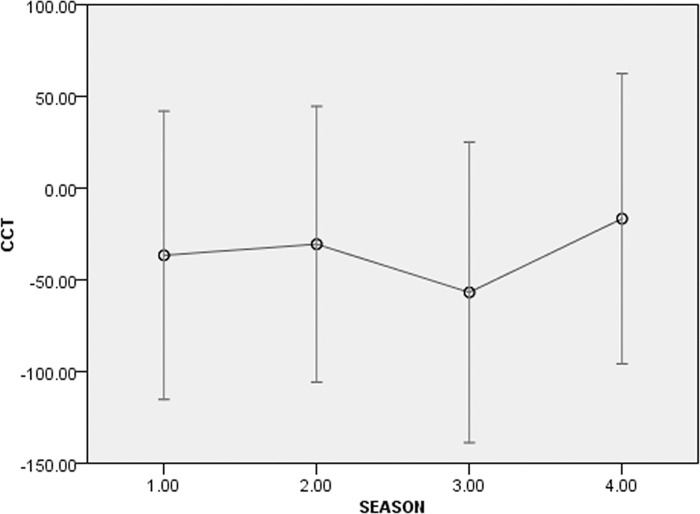
SPSS 20.0 output bar graph showing estimated means of Corrected Conciliatory Tendency (CCT, %) for the significant effect (season: 1 = lactation; 2: pre-mating; 3 = mating; 4 = pregnancy), for group C_c_. Season is the only factor that significantly influences the CCT distribution in the study groups (GLMM; F = 1.674, df1 = 15, df2 = 36, p = 0.102). The conciliatory tendency % is lowest during mating.

Of all the GLMM models tested for group C_c_ (AICc range = 393.675–534.649), the best one was the full model (Intercept: F = 3.103, df1 = 15, df2 = 38, p = 0.002), including the combination of individual features (sex*rank: F = 1.448, df1 = 1, df2 = 38, p = 0.236; age*rank: F = 0.849, df1 = 1, df2 = 38, p = 0.363), individual identity (F = 1.805, df1 = 9, df2 = 38, p = 0.099), and the season (lactation, pre-mating, mating, and pregnancy; F = 3.844, df1 = 3, df2 = 38, p = 0.017). Fig 5 shows the output for the best model. Again, two individuals accounted for part of the CCT variation but only the season had a significant effect on the distribution of CCTs throughout the year, with CCT values being minimum during the mating season (Figs 5 and 6). Both in captivity and in the wild, males (M_in_) and females (F_in_) initiated the first affinitive contact with comparable frequencies in all seasons (captivity, range: M_in_ = 47,22–51.72%; F_in_ = 48.28–52.77%; wild, range: M_in_ = 46,88–50.00%; F_in_ = 50,00–60,00%).

## Discussion

Reconciliation was present both in the wild and in captivity (prediction 1 supported), and specifically in two out of five wild troops of *L*. *catta* (Fig 2) and in two captive troops (group C_c_, present study; group A_c_, [65]) (Fig 1). When considering either all the study groups or group C_c_ only (for which longitudinal data were available), season was the only effect that significantly influenced the fluctuation in the frequency of reconciliation events (Figs 3 and 5). In particular, the conciliatory tendency was lowest during the mating season (prediction 2 supported; Figs 4 and 6).

Reconciliation was found in another despotic species with linear hierarchy, the wolf (*Canis lupus*; mean conciliatory tendency, 44.1% in the wild [11]; 53.3% in captivity [5]). In wolves, each group defends its own territory as a unit [118]. Yet, even if the alpha male normally guides the movements of the wolf pack and initiates aggressions against intruders [118], the subordinate members can sometimes oppose their leader’s actions. According to Zimen [119], no subject decides alone the carrying out of activities that are vital to the group cohesion. In short, wolves are highly despotic but also extremely cooperative. The existence of an extremely cooperative pack has presumably to do not only with hunting but also with the collective rearing of offspring and, consequently, with reproductive success [120]. Probably, in wolves the benefit of preserving the social bonds through reconciliation outweighs the cost of pack disruption, which would be detrimental for both dominants and subordinates. Thus, reconciliation can be found in despotic groups provided that they show some form of cooperation [51]. Further evidence of this assumption is the presence of reconciliation in spotted hyenas (*Crocuta crocuta* [53]). Hyenas are despotic but often depend on the help from other group members during hunts, defence of ungulate carcasses against competitors, and coalition formation that is important in both the acquisition and maintenance of social rank [53]. Cooperation and despotism are two opposite forces that contribute in shaping reconciliation patterns, as it becomes especially clear when comparing species differing only in some aspects of the social system. In hyenas, as in wolves, the necessity to cooperate overcomes the competition between dominants and subordinates, which explains the presence of reconciliation. The lower levels of reconciliation observed in hyenas (mean conciliatory tendency: 11.3% [53]) may be due to the fact that, contrary to wolves, spotted hyenas live in a fission fusion society allowing dispersal (other than reconciliation) as an exit strategy. The influence of the cooperation pressure over the suitability of engaging in reconciliation is even more evident when comparing spotted hyenas with ring-tailed lemurs. Although both species possess steep female dominance, they strongly differ in the level of cooperation. Unlike hyenas, cooperation in *L*. *catta* is limited to the coalitionary support provided to the dominant female by other females during targeted aggression toward conspecifics (to defend territory boundaries or to evict them from the group or the core area of the home range; [10], [58], [121]). This limited cooperation can explain why *L*. *catta* show the lowest conciliatory tendencies (9–10%). In some macaque species, it has been observed that the higher the cooperation and tolerance levels, the higher the reconciliation rates [52]. This principle can be extended to include other primates. For example, conciliatory tendencies can reach more than 40% in bonobos (*Pan paniscus*) and crested macaques (*Macaca nigra*) [22], [122] and plummet to less than 15% in more despotic and less cooperative species such as Assamese macaques (*Macaca assamensis*) and western gorillas (*Gorilla beringei*) [25], [123]. Of course, the distinction between more and less cooperative species is not always clear cut because primates can form rather complex societies and the individuals of certain subgroups can be more cooperative than the group as a whole, as occurs when cooperative breeding, matriline support, or brotherhood coalitions are in place [124].

Although at low levels, reconciliation seems to be possible in despotic species like *L*. *catta* when the cooperation-competition balance tilts in favor of cooperation because the benefits of peace making overcome the costs of leaving conflicts unmanaged. But when reproduction is at stake, as it is in lemurs during the once-a-year mating period, both male-female and male-male competition is too high [58],[125] for conflicts to be peacefully resolved. In our study we found that aggression in the mating period was particularly high between males and between females and males (with females initiating the aggression). Consistently, conciliatory rates of both males and females were minimal in the mating season (Figs 4 and 6) likely because in this period the behaviors of individuals are oriented toward reproduction more than maintenance of social stability. Based on these results, it is possible to assert that reconciliation is season-dependent in *L*. *catta*. Sex was not the explaining variable for the observed fluctuations in conciliatory tendencies. Consistently, both males and females initiated the post-conflict reunion with comparable frequencies throughout the year.

The only study to date that has investigated the seasonal fluctuations of reconciliation in another despotic primate species [84] reported that in female Japanese macaques (*Macaca fuscata*) mating—and not other factors such as changes in activity budgets and dietary composition—had profound effects on peace-making. In fact, the conciliatory tendency–informing reconciliation rates—was significantly lower during the mating season than the non mating season [84]. The authors commented that the negative effects of the mating season on reconciliation within female Japanese macaques may be due to the relevance of female competition for access to male partners in multimale, multifemale societies characterized by adult male dominance. In *L*. *catta* the situation is reversed: adult females are dominant over males [57–59] and the competition and stress levels during the mating period are highest among males trying to gain access to female partners [125]. Despite the difference in the dominant sex between *L*. *catta* and *M*. *fuscata*, the result is similar: reconciliation is lowest during the extremely competitive mating period.

A possible explanation for the seasonal distribution of reconciliation can lie in how hormones modulate the propensity to affiliate with others, and consequently to reconcile. It is worth remembering that the very definition of reconciliation implies the use of affinitive contacts for the purpose of peace making [20]. As well as in other animals in which the sexual context is associated with aggression and competition [126], [127], *L*. *catta* males experience the highest levels of testosterone during the extremely high competitive mating period [128], which also coincides with the lowest levels of inter-male affiliation [91]. The stress hormones may also increase as a result of aggression, eliciting the fight or flight response [129] and therefore leaving little space for post-conflict affiliation among males. However, literature reports contrasting results on the level of stress hormones (fecal glucocorticoid) in *L*. *catta* males during the mating period [125].

Besides male affiliation, the high levels of estradiol associated with the mating period can reduce affiliation between primate females, for example in rhesus monkeys (*Macaca mulatta* [130]). Additionally, in human and non human primates, other hormones such as oxytocin and prolactin may influence female affiliation levels throughout the year because they can enhance individual propensity to affiliate and are higher in non-mating periods [131–138]. Consistently, *L*. *catta* females (aggressors) mainly initiated conciliatory affiliation in group A_c_ [65]. Therefore, hormonal influence may partly explain the variation in post-conflict conciliatory affiliation across the year.

The seasonality of the conciliatory tendency in *L*. *catta* documented in the present study is also consistent with the variation of inter-male affiliation rates recorded by Gabriel, Gould & Kelley [91] in the same species, at four sites of Madagascar. These authors observed that inter-male affiliation levels varied across reproductive periods, with the lowest frequencies occurring during the mating period. Overall, the seasonal fluctuations of the reconciliation tendency observed in *L*. *catta* appear to be sustained by both physiological and socio-ecological data.

Access to females is not the only item worth competing for. Another valuable resource connected to reproductive success is offspring. We observed that in both the wild and captivity female-female aggression was highest during pregnancy and during the lactation period (Table 3), when the newborn is still carried out by the mother. It has been hypothesised that dominant females may target subordinate ones to prevent them from conceiving or to cause them to lose their infants because subordinate females with vital offspring can potentially acquire a higher ranking status in the social group and subtract resources [58], [121]. Food also represents a valuable commodity for the members of social groups, eliciting competition more than cooperation. Consistently, in the wild, reconciliation was found in a group of *Eulemur rufus x collaris* and in two groups of *Propithecus verreauxi* but never in the feeding context [45], [73]. This situation reinforces the idea that when a valuable resource is concerned and cooperation is low (e.g. mate for reproduction, high energy food), gaining access to that resource can be more rewarding than repairing the relationship with a former opponent in the short term, via post-conflict reunions. As a future direction, it would be interesting to assess if and how conciliatory tendencies of *L*. *catta* are influenced by the context and the individuals involved in the conflicts within each season. We expect that post conflict reunions are lowest in competive contexts (e.g. feeding) and between competing individuals (e.g. females during pregnancy and lactation; males during mating, etc.).

In conclusion, we posit that the ability to reconcile is expressed whenever the benefits of intra-group cooperation overcome the costs of competition, as occurs when a limited, wanted resource is at stake. In summary, this study shows that in despotic social groups in which coalitions are observed, the right question is not *if* but *when* reconciliation can be present.

## Supporting Information

S1 Dataset Dataset used to investigate the occurrence and seasonality reconciliation in *Lemur catta*. (XLSX)

S1 Fig Full size version of Fig 3. (TIF)
